# The genetic landscape of 5T models for multiple myeloma

**DOI:** 10.1038/s41598-018-33396-w

**Published:** 2018-10-09

**Authors:** Ken Maes, Bram Boeckx, Philip Vlummens, Kim De Veirman, Eline Menu, Karin Vanderkerken, Diether Lambrechts, Elke De Bruyne

**Affiliations:** 1 Department of Hematology and Immunology, Myeloma Center Brussels, Vrije Universiteit Brussel, Brussel, 1090 Belgium; 2 Laboratory for Translational Genetics, Department of Human Genetics, Katholieke Universiteit Leuven, VIB Center for Cancer Biology, Leuven, 3000 Belgium; 3 Department of Clinical Hematology, Ghent University Hospital, Gent, 9000 Belgium

## Abstract

Murine models for multiple myeloma (MM) are often used to investigate pathobiology of multiple myeloma and disease progression. Unlike transgenic mice models, where it is known which oncogene is driving MM disease, the somatic aberrations of spontaneous syngeneic 5T models of MM have not yet been reported. Here, we analyzed the copy-number alterations (CNA) and mutational landscape of 5T2, 5T33vv and 5TGM1 murine MM models using whole-genome and whole-exome sequencing. Forty four percent of the genome of 5T2 cells is affected by CNAs while this was only 11% and 17% for 5T33vv and 5TGM1 cells, respectively. We found that up to 69% of the genes linked to gain of 1q or deletion of 13q in MM patients are present as respectively gains in 5T2 cells or deletions in 5T33 and 5TGM1 cells. Exome sequencing furthermore revealed mutations of genes involved in RAS/MAPK, PI3K/AKT1 and JAK/STAT signaling, DNA damage response, cell cycle, epigenetic regulation and extracellular matrix organization. We observed a statistically significant overlap of genes mutated in the 5T models and MM patients. Overall, the genetic landscape of the 5T models is heterogeneous with a high number of aberrations involving genes in various multiple myeloma-related pathways.

## Introduction

Multiple myeloma (MM) is a plasma cell malignancy characterized by the accumulation of plasma cells in the bone marrow (BM). In the past years, deciphering the genetic and epigenetic landscape of MM has identified which pathways and genes drive myelomagenesis. Gene expression profiling identified molecular subclasses of MM patients which correlate with prognosis and drug sensitivity^1,2^. Moreover, progression of MM is linked to alterations in DNA-methylation profiles^3^. Next-generation sequencing (NGS) uncovered the heterogeneity of genetic alterations, clonal patterns and mutational signatures in MM patients^4–8^. These studies may lead to new targeted- and risk adapted-treatments.

Murine MM models are used to study the pathobiology of MM and validate therapeutic targets. The models can be categorized in spontaneous, induced, transgenic and xenograft models^9^. Xenograft models involve the engraftment of human MM cells in immunodeficient SCID mice. Transgenic models include the Eµ-XBP1s and Eµ-MAF where respectively XBP1s and MAF are under the control of the immunoglobulin enhancer and drive the development of MM^10,11^. A more recent transgenic model is the Vk*MYC model, in which the oncogene MYC becomes overexpressed by AID-dependent somatic hypermutation in post-germinal center B-cells of transgenic mice. This model develops MM with similar characteristics as the human disease and is used for studying MM (immune-)pathobiology and pre-clinical drug testing^12–14^. Induced syngeneic immunocompetent models include the MOPC models which are induced by the injection of mineral oil or pristane^15,16^. However, these plasmacytomas typically grow extramedullary. Recently, a MOPC line that homes and grows in the bone marrow has been derived (MOPC-315.BM)^17^. Spontaneous syngeneic immunocompetent models of MM include the 5T models derived from C57BL/KaLwRij mice. This inbred strain spontaneously develops benign monoclonal gammopathy, similar to monoclonal gammopathy of unknown significance in humans. Benign monoclonal gammopathy progresses into B-cell malignancies, including MM with a frequency of 0.5% in mice older than 2 years. Several 5TMM models were developed by serial transplantation of the BM of diseased mice into young syngeneic mice^18^. These models closely resemble human MM disease as MM cells are localized in the BM where they produce large amounts of serum M-protein. Moreover, the diseased BM displays enhanced angiogenesis and increased bone resorption^19^. The best studied models are the 5T33vv and 5T2 model, both growing exclusively *in vivo*. Two BM stroma-independent murine cell lines were derived from 5T33vv cells namely the 5T33vt^20^ and 5TGM1 cells^21^. The 5TMM models have been extensively used to study MM pathobiology and for evaluating existing and novel therapies^22–27^.

The genetic basis for the predisposition of C57BL/KaLwRij mice to develop MM is currently under investigation. Using gene expression profiling, Noll *et al*. described the deletion of SAMSN1 in C57BL/KaLwRij mice compared to C57BL/6. They further demonstrate that SAMSN1 re-expression in 5TGM1 cells prevents MM formation^28^. This was confirmed by whole-genome sequencing (WGS) and whole-exome sequencing (WES) of C57BL/KaLwRij mice indicating that SAMSN1 deletion is important for MM predisposition of C57BL/KaLwRij mice and that SAMSN1 is a potential tumor suppressor gene^29^. To date, however, the mutational profile and copy number instability has not been investigated in the 5TMM models derived from the C57BL/KaLwRij mice. To address this issue, we performed low coverage WGS and WES of 5T33, 5TGM1 and 5T2 myeloma cells. Understanding these aberrations in the 5TMM models may be exploited to identify new biomarkers or address the functional consequences in terms of drug response and disease progression.

## Results

### Identification of copy number alterations in the 5TMM models

**Figure 1:**
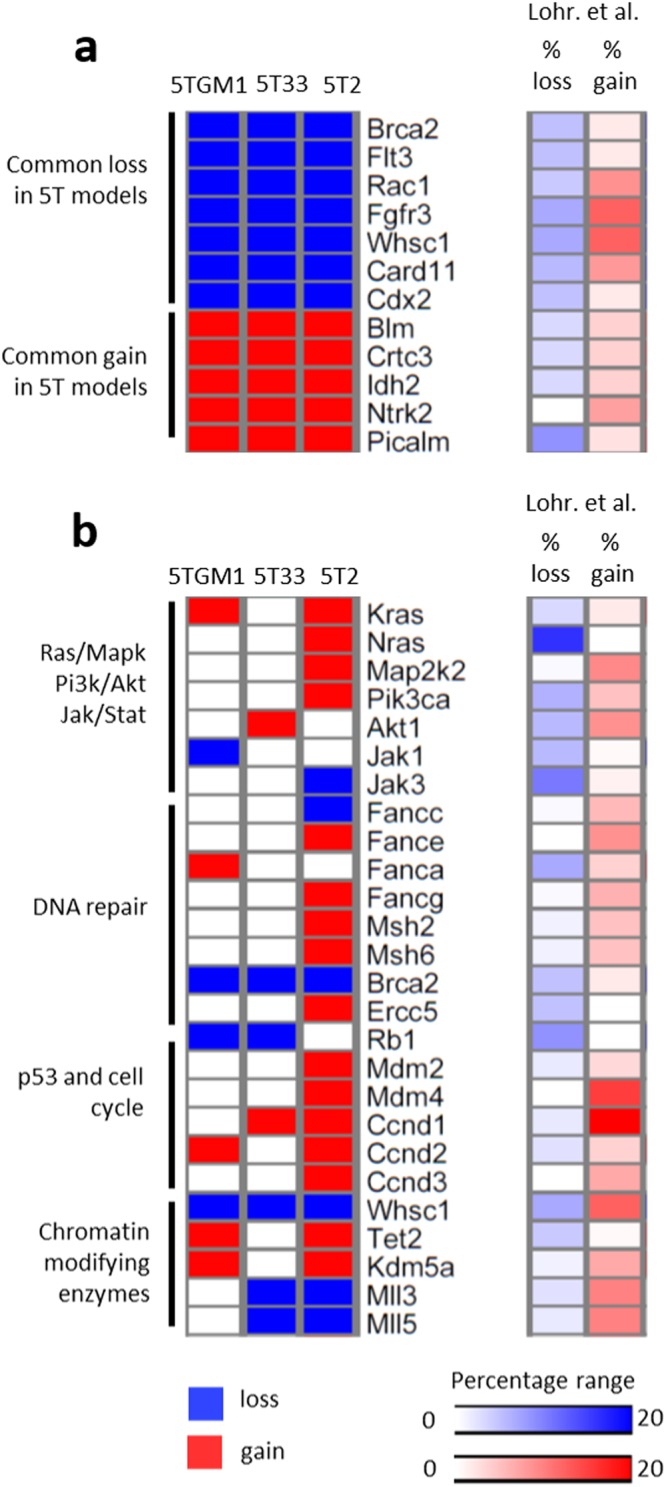
Summary of pathways affected by copy number alterations in the 5T models and the comparison with human MM patients. (**a,b**) Heatmap of genes with copy number alterations in the 5T models and the frequency of copy number alteration in human MM patients of the corresponding human orthologue genes. Blue indicates loss and red indicates gain of the particular gene on DNA level. The frequency (percentage range) of copy number alterations in human MM patients was retrieved from the supplementary data published by Lohr *et al*.^4^ (Related to Supplementary Tables 2–4).

We first performed low-coverage WGS to analyze copy number alterations (CNAs) in 5T33vv, 5TGM1 and 5T2 cells for which the metrics can be found in Supplementary Table 1. As controls, we used germline DNA from the background mouse strain C57Bl/KaLwRij, which is a substrain of the C57Bl/6 mouse strain^29^. The 5TMM samples displayed focal as well as whole-arm CNA compared to normal germline DNA of C57Bl/KaLwRij and C57Bl/6. The full list of CNAs is present in Supplementary Table 2. Supplementary Figs S1 and S2 show the copy number profiles of the tested 5T models and germlines, respectively. We calculated the fractions of aberrant regions in each 5TMM model. 5T2 cells contained the largest degree of aneuploidy compared to 5TGM1 and 5T33vv (44% vs. 17% and 11%, respectively). In agreement with their subclonal relation, 6% of the genomic aberrant regions was shared between 5TGM1 and 5T33vv cells, while 11% and 5% was unique, respectively (Supplementary Table 1). All three models show the loss of a regions on chromosome 5 encompassing *Brca2, Flt3, Rac1, Card11, Cdx2, Fgfr3 and Whsc1*. A common amplified region is detected involving chromosome 7 and includes *Blm, Ctcr3, Idh2, Ntkr3 and Picalm* (Fig. 1a and Supplementary Fig. S1). The corresponding frequency of CNAs in the MM patients was retrieved from the supplementary data published by Lohr *et al*.^4^ and are also shown in Supplementary Table 3.

### The 5TMM models contain CNAs that affect genes linked to growth factor signaling and extracellular matrix organization

To delineate potential cancer-related CNAs, we identified cancer consensus genes (according to COSMIC database)^30^ which are highlighted in yellow in Supplementary Table 2 and of which the most interesting are displayed in the CNA plots (Supplementary Fig. S1). Next, we used Cytoscape to analyze pathway enrichment of genes affected by CNAs and results are provided in Supplementary Table 4. The identified pathways mainly have a role in growth factor signaling including PI3K/Akt and Ras signaling as well as cell cycle progression. Pathways linked with cell junctions were also enriched, including focal adhesion, extracellular matrix organization and Rap1 signaling pathways. In Fig. 1b, we provide an overview of the most interesting genes with copy number alteration and the involved pathways. The corresponding frequency of CNAs in the MM patients^4^ are also illustrated (Fig. 1b, Supplementary Table 3). Briefly, 5T2 cells contained several focal amplifications encompassing oncogenes including *Kras*, *Nras, Map2k2* and *Pik3ca*. *Kras* and *Akt1* was also focally amplified in the 5TGM1 and 5T33vv model respectively. *Jak1* and *Jak3* showed focal copy number loss in 5TGM1 and 5T2, respectively. Several CNA regions also contain genes involved in DNA damage response (Fig. 1b). We observed a focal deletion of the tumor suppressor *Brca2* involved in homologous recombination in the 5T33vv and 5TGM1 models, while chromosome 5 (including *Brca2*) was fully deleted in 5T2. A Fanconi anemia gene, *Fancc*, showed copy number loss in the 5T2 model. This model also showed an amplification of *Fance, Fancg* and mismatch repair genes *Msh2* and *Msh6*. Genes involved in cell cycle regulation were also affected. Most interestingly, we detected a focal deletion on chromosome 14 encompassing the tumor suppressor *Rb1* in 5TGM1 and 5T33vv. Moreover, at least one member of the oncogenic cyclin D family was focally amplified in all models. The oncogenes *Mdm4* and *Mdm2*, two negative regulators of tumor suppressor gene *Trp53*, showed a gain in the 5T2 model as part of large scale amplifications on chromosomes 1 and 10, respectively. Lastly, CNAs in genes with epigenetic functions included the loss of *Whsc1* in all models, gain of *Tet2* and *Kdm5a* in 5T2 and 5TGM1, gain of *Brd4* and *Kdm5a* in 5T2 and loss of *Mll3* and *Mll5* in 5T2 and 5T33vv (Fig. 1 and Supplementary Fig. S1). In conclusion, genes located in the affected regions are involved in growth factor signaling pathways as well as organization of the micro-environment. Moreover, we identified CNAs of genes known to be implicated in myelomagenesis.

### The mutational spectrum in the 5TMM models

We next investigated the mutational landscape of the 5TMM models by WES which was performed on the 5T33vv, 5TGM1 and 5T2 model and their matched germlines, i.e. C57Bl/KaLwRij and C57Bl/6. The exome metrics can be found in Supplementary Table 5. An overview of the mutations of the two tested strain germlines compared to the reference genome Mm10 is summarized in Supplementary Table 6. To identify tumor specific mutations, we compared the mutations in the murine models with those in the two matched germlines. We identified 389, 534 and 534 substitutions (including 314, 417 and 407 non-synonymous substitutions) and 24, 8, and 14 indels in the 5T2, 5T33vv and 5TGM1 models, respectively, which are shown in Supplementary Table 7. Hundred and nine shared mutations were found between the related models 5T33vv and 5TGM1, this is 26 and 27% of the total number of mutations for the 5T33 and 5TGM1, respectively.

### Overview of the mutational signatures in the 5TMM models

**Figure 2:**
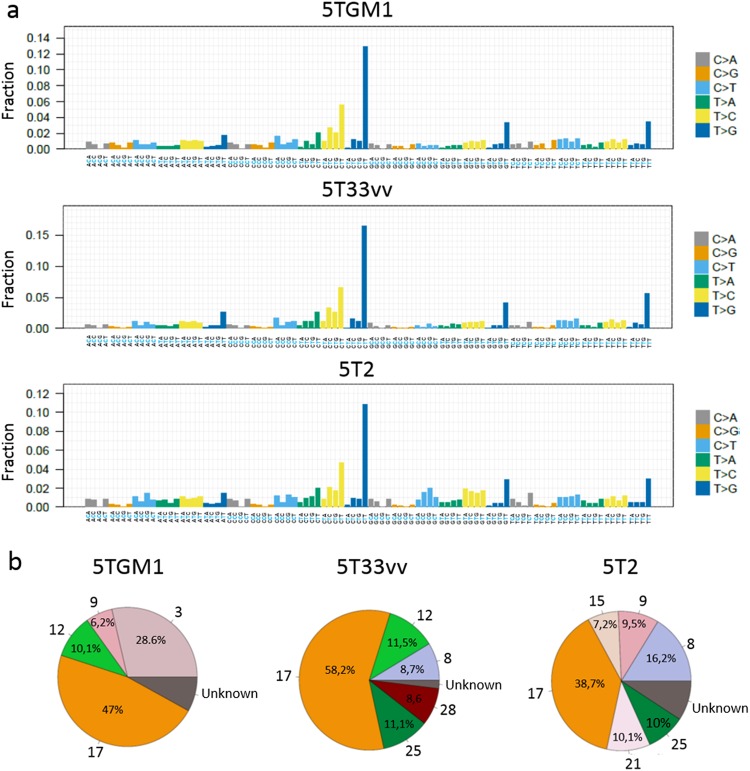
Mutational signatures in the 5T models. The mutational signature of the different 5T models was calculated according to the database of mutational signatures in COSMIC. (**a**) Overview of the fraction of observed mutations in the context of the preceding and following base. (**b**) Pie plots of the relative contribution of mutational signatures for each of the tested 5T models.

Next, we generated mutational signatures (according to COSMIC database)^30^ to investigate which type of mutations are dominant in the 5TMM models (Fig. 2). Mutational signature 17 was the dominant mutational signature in the tested 5T models. It represented 48.7%, 58.2% and 38.7% of the entire signature in 5TGM1, 5T33vv and 5T2 respectively. Signature 17 is found in several cancer types, including B-cell lymphoma, breast and lung cancer and contains T > G mutations in the context of NTT. Common signatures found in 5T2 and 5T33vv were signatures 8 and 25. Signature 8 is found in medulloblastoma, breast cancer and recently also in MM but it has no known etiology^30,31^. Signature 25 has been found in Hodgkin-lymphoma and has also no known etiology. Signature 9 was shared in 5T2 (9.5%) and 5TGM1 (6.2%). This signature is found in chronic lymphocytic leukemia, B-cell lymphoma and recently also in MM^30,31^ and is linked to polymerase η activity during activation-induced cytidine deaminase (AID)-mediated somatic hypermutation. Signature 12, present in liver cancer, was commonly found in 5TGM1 and 5T33vv (10.1% and 11.5% respectively). In addition, signature 3 is unique for 5TGM1 where it represents 28.6% of all mutations. This signature is linked to defects in homologous recombination-mediated repair of double strand breaks. Signature 21 (10.1%) and 15 (7.2%) are unique for 5T2, while signature 28 (8.6%) is unique for 5T33vv (Fig. 2). Signature 21 and 28 were found in stomach cancer but have no known cause. Signature 15 is linked to defects in DNA mismatch repair^30^.

### Potential oncogenic mutations in the 5TMM models

**Figure 3:**
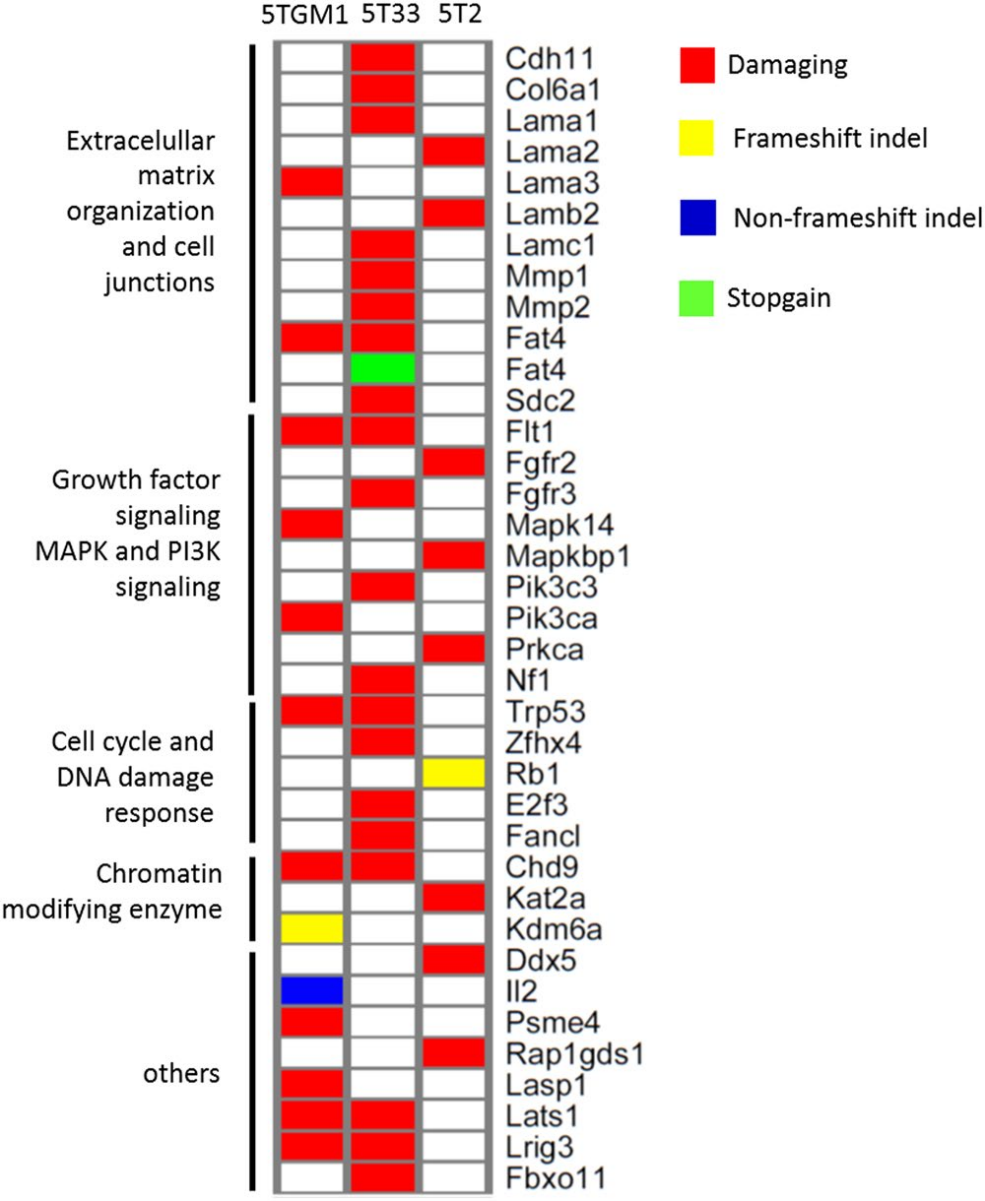
Overview of the mutational landscape of the tested 5T models. Heatmap of mutated genes in the 5T models. Only non-synonymous damaging mutations were considered. PROVEAN and Sift were used to predict the effect of the mutations on protein functionality. This is indicated by the color legend (Related to Supplementary Tables 7 and 8).

We next performed pathway enrichment analysis on the somatic mutations using Cytoscape (Supplementary Table 8). Enriched pathways were linked with extracellular matrix organization and cell junctions including integrin, Rap1 and cadherin signaling. We also found an enrichment of PI3K, Wnt and growth factor-related signaling. These results are in line with the pathways identified based on the CNAs. Because of the limited number of different models, we could not apply stringent statistical tools to investigate recurrent potential driver genes. Instead, we selected the non-synonymous mutations followed by Provean and Sift analysis to predict whether a given mutation results in a damaged protein function. The most interesting genes and involved pathways are summarized in Fig. 3. Damaging mutations in genes involved in extracellular matrix organization and cell junctions were most prominent in 5T33vv cells. Mutations were found *in Cdh11* (p.N300K), *Col6a1* (p.E594A)*, Lama1* (p.D94A)*, Lamc1* (p.T900A)*, Mmp1* (p.324T)*, Mmp2* (p.S64C)*, Fat4* (p.E965D and p.R26X (stopgain)) and *Sdc2* (p.F163S). 5T2 cells contained mutations in *Lama2* (p.L503V) and *Lamb2* (p.V133F), while 5TGM1 cells contained mutations in *Lama3* (p.E1592D) and *Fat4* (p.E965D). We also found mutations in genes involved in growth factor signaling including MAPK and PI3K signaling. For 5T33vv, we found mutations in *Fgfr3* (p.V87A), *Pik3c3* (p.L493V) and *Nf1* (p.E106A). 5TGM1 displayed mutations in *Pik3ca* (p.L387V) and *Mapk14* (also known as p38α; p.F59V). For 5T2, we found mutations in *Fgfr2* (p.S239R), *Mapkbp1* (p.K807M) and *Prkca* (p.E225G) (Fig. 3). Regarding cell cycle regulation and DNA damage response, we found that 5T2 cells contain a frameshift deletion in *Rb1*. 5TGM1 and 5T33vv cells contained a *Trp53* mutation (p.K117M), while the latter also showed a mutation in *E2f3* (p.V310M), *Zfhx4* (p.L1750P) and *Fancl* (p.F36L). Finally, chromatin modifying enzymes showed mutations. 5TGM1 and 5T33vv cells harboured a mutation in the chromodomain helicase *Chd9* (p.H170Y). 5TGM1 cells had a frameshift deletion in the lysine demethylase *Kdm6a*. 5T2 cells showed a mutation in the lysine acetyltransferase *Kat2a* (T543A) (Fig. 3).

### Differential dependence of murine MM cells on MAPK and PI3K pathways

**Figure 4:**
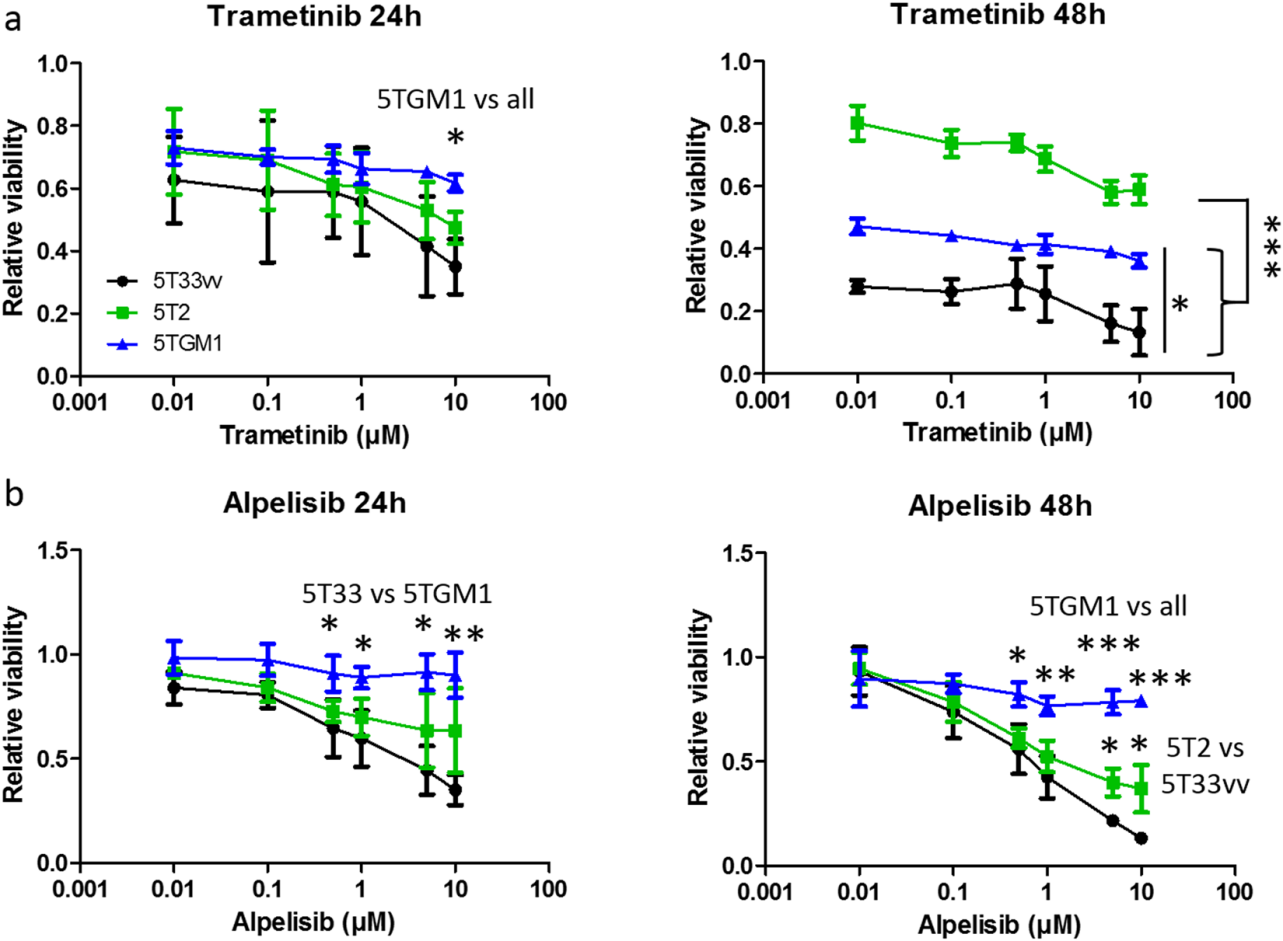
Sensitivity of murine MM cells to compounds targeting mutated pathways. Primary MM cells (5T33vv and 5T2; n = 3) and 5TGM1 cells (n = 4) were treated with indicated concentrations of the compounds for 24 and 48 hours. Cells were treated with MEK1/2 inhibitor Trametinib (**a**) and a PI3Kα inhibitor Alpelisib (**b**). Cell viability was determined by Cell-Titer-Glo assay with technical replicates in triplicate. Results represent mean and SD from at least three independent experiments. Statistical analysis was done by one-way ANOVA followed by Tukey’s multiple comparison test. * represents p < 0.05, ** indicates p < 0.01, *** indicates p < 0.0001. (Related to Supplementary Table 9).

Given the observed genetic alterations in MAPK and PI3K signaling in all three models, we investigated whether these murine MM cells are dependent on MAPK and PI3K pathways for survival. Mutations and copy number alterations in MAPK pathway were found on the level of MAPK activators and downstream MAP3K, MAP2K, MAPK genes (Figs 1 and 3, Supplementary Tables 2 and 7). To interfere with MAPK signaling, we used trametinib (a Mek1/2 inhibitor) and assessed the sensitivity of the murine MM cells. After 48 h, a clear difference in sensitivity was noted with the 5T2 cells being the least sensitive compared to 5TGM1 and 5T33vv cells (Fig. 4a, Supplementary Table 9). Concerning the PI3K pathway, we observed mutations and copy number alterations in catalytic and regulatory subunits of PI3K-related genes (Figs 1 and 3, Supplementary Tables 2 and 7). We interfered with PI3K using the PI3Kα inhibitor alpelisib. The 5T33vv and 5T2 cells were more sensitive to inhibition of PI3K compared to 5TGM1 cells (Fig. 4b, Supplementary Table 9). The IC50 at 48 h is 0.7 µM and 1.5 µM for 5T33vv and 5T2 respectively, while it was not reached for 5TGM1 cells. These results indicate a difference in dependency on the PI3K and MAPK pathways between the 5TMM models.

### The 5TMM models contain CNAs affecting genes linked with gain of 1q and loss of 13q in MM patients

**Figure 5:**
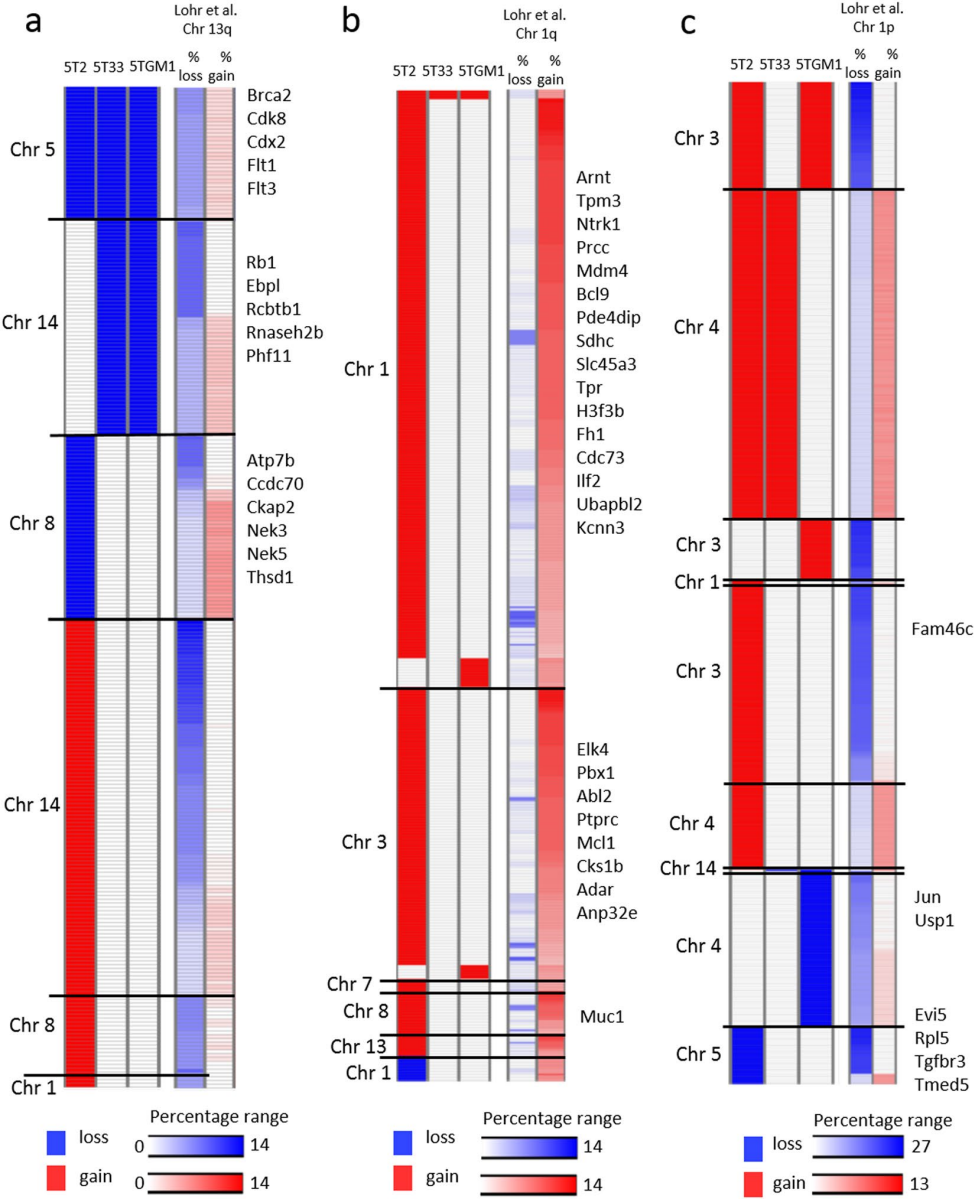
Overlap of the CNAs in the 5TMM models and MM patients. (**a**) Heatmap of copy number alterations in genes on human chromosome 13q and their corresponding murine orthologue. (**b**) Heatmap of copy number alterations in genes on human chromosome 1q and their corresponding murine orthologue. (**c**) Heatmap of copy number alterations in genes on human chromosome 1p and their corresponding murine orthologue. The frequency of copy number alterations in human MM patients was retrieved from the supplementary data published by Lohr *et al*.^4^ (Related to Supplementary Table 3).

In MM patients, CNA are often detected and are associated with prognosis^32^. In particular, gain of 1q and deletion of 1p,13q and 17p are associated with poor prognosis^7,32–34^. We investigated whether the tested 5TMM models contain CNAs which affect genes known to be part of CNAs in patients. Therefore, we made an overlap of the genes affected by CNAs in patients^4^ and the corresponding murine orthologs genes affected by CNAs in the 5TMM models (Supplementary Table 3). Interestingly, 66.4% and 69.6% of genes implicated in gain of 1q or deletion of 13q in patients, respectively, were found to be part of CNA in the 5TMM models. The overlap for deletion 1p was 48.4%, while no overlap for deletion 17p was found. As mentioned above, we observed an overlap of 69.6% for deletion 13q corresponding to 257 genes (Fig. 5a). Out of the 257 genes, 35 genes located on murine chromosome 5, were commonly deleted in all three 5TMM models. Fifty-six genes, located on murine chromosome 14, showed loss of copy number in the 5TGM1 and 5T33vv cells. Twenty four out of these 56 genes are linked to del13q without evidence of copy number gain of these genes in MM patients^4^. Amongst them are *Rb1*, *Ebpl*, *Rcbtb2* and *Rnaseh2b*, four genes suggested to be important in MM patients for their link to poor prognosis^33,34^. In addition, the 5T2 cells contained 48 genes on chromosome 8 that show loss of copy number. The remaining 122 genes are part of copy number gains on chromosomes 14, 3 and 1 in the 5T2 cells only (Fig. 5a). For gain of 1q, we observed an overlap of 66.4% corresponding to 704 genes (Fig. 5b). In fact, 5T2 cells contain 643 genes that are part of copy number gains on murine chromosomes 1, 3, 7, 8 and 13. Of these 643 genes, 267 genes are linked to gain of 1q in patients without evidence of copy number loss of these genes in MM patients^4^. Within these 267 genes, we observed genes suggested to drive the poor prognostic feature of gain of 1q including *Cks1b*, *Ilf2*, *Adar*, *Anp32e*, *Ubap2l*, *Kcnn3* and *Arnt* (Fig. 5b)^33,34^ (Jin *et al*., 22nd EHA conference 2017; abstract LB260). Concerning deletion 1p, the overlap was 48.4% which equals 560 genes (Fig. 5c). Of these 560 genes, 36, 97 and 1 gene(s) were part of copy number loss in 5T2, 5TGM1 and 5T33vv cells respectively. In 5T2 cells, 30 genes out of 36 are linked to deletion of 1p in patients without evidence of copy number gain of these genes in patients^4^. These include *Evi5, Tgfbr3, Tmed5 and Rpl5*. In 5TGM1 cells, 24 genes out of 97 are linked to deletion of 1p in patients without evidence of copy number gain of these genes in patients^4^. In conclusion, the identified CNAs in the 5T models involve genes known to be altered by CNAs in MM patients which are linked with poor prognosis^32^.

### Overlap of the mutational spectrum in the 5TMM models and MM patients

In MM patients, several recurrent mutated genes are involved in the MAPK pathway (*NRAS, BRAF, KRAS*), NFKB pathway (*TRAF3, CYLD* and *LTB*) and DNA repair and cell cycle regulation (*TP53, ATM, ATR, RB1, CCND1*). Other recurrent mutated genes include *PRDM1, SP140, EGR1, IRF4* and *FGFR3*^4,5,32,35^. In the 5T models, we observed *Trp53* mutations in 5TGM1 and 5T33vv while *Rb1* was mutated in 5T2. Moreover, a mutation in *Fgfr3* was found in the 5T33vv model (Supplementary Table 7). We next assessed the similarity of the mutational landscape of the 5TMM models with that of patients. Because of the subclonal relation between the 5TGM1 and 5T33vv samples, we included only those genes that are mutated in the 5T33vv and the 5T2 murine models (Supplementary Table 7). The presence of these mutated genes was compared to that of MM patients’ of two large datasets published by Lohr *et al*. (n = 203) and Walker *et al*. (n = 463)^4,5^. The overlap was statistically significant between genes mutated in both murine models (n = 28) and the genes mutated in >2% or >3% of the MM patients (p < 1E-8). Likewise, the overlap was also significant for all genes mutated in one of the murine MM models (n = 886, P-value < 1E-9) (Table 1). Genes mutated in both datasets of MM patients include *Trp53*, *Zfhx4*, *Fgfr3*, *Fat3, Fat4* and *Lrp1b*. Other overlapping genes mutated in one dataset include *Nf1*, *Robo1* and *Rb1*. In conclusion, we found a statistically significant overlap of mutated genes in the 5TMM models and the human situation with *Trp53* and *Rb1* being the most interesting.

**Table 1:** Comparison between mutational landscape of murine and human multiple myeloma samples.

Recurrent mutations in the 5T mouse models				
Overlap with	Overlap with the 28 recurrent mutated genes in murine myeloma	Theoretical expected overlap	Fold-change of the enrichment in the murine tumors	p-value
Lohr *et al*. (>3% mutated)	6	0.15	40.4	7.10E-09
Lohr *et al*. (>2% mutated)	9	0.45	20.2	2.90E-10
Walker *et al*. (>3% mutated)	5	0.07	75.5	6.60E-09
Walker *et al*. (>2% mutated)	7	0.14	50.1	7.50E-11
**All overlapping mutations in the mouse models**				
**Overlap with**	**Overlap with the 886 mutated genes in murine myeloma**	**Theoretical expected overlap**	**Fold-change of the enrichment in the murine tumors**	**p-value**
Lohr *et al*. (>3% mutated)	31	5.27	5.9	1.20E-14
Lohr *et al*. (>2% mutated)	59	15.8	3.7	<E-16
Walker *et al*. (>3% mutated)	18	2.35	7.7	6.90E-11
Walker *et al*. (>2% mutated)	23	4.95	4.6	2.80E-09

## Discussion

In this study, we describe for the first time somatic aberrations on a genome wide level in a murine model for MM. Concerning the CNAs identified in the 5TMM models, the 5T2 model showed the largest degree of aneuploidy compared to 5T33vv and 5TGM1. Previous karyotyping results identified several chromosomal aberrations in 5T models^36^. In agreement with our findings, a larger amount of chromosomal aberrations was found in 5T2 compared to 5T33vv as well as the loss of a sex chromosome. Nevertheless, our sequencing approach is more sensitive to detect the more focal changes compared to the previous study. A common copy number gain for all tested models was found for at least one cyclin D member which have also been detected in patients^4^. Cyclin D dysregulation may be a key event in these models which is similar to the human situation^37^. Two other key events in the models may be the common loss of a region encompassing *Brca2* and *Whsc1* and the common gain of a region encompassing *Blm, Idh2* and *Picalm*. Of interest, the 5T2 model also shows gain of the same genes involved in gain of 1q in patients including *Cks1b and Mcl1* amongst others. Likewise, the 5TGM1 and 5T33vv model show a loss in chromosome 14 affecting genes associated with deletion of 13q in MM patients, of which *Rb1* is the most important one. 5TGM1 and 5T2 also show CNAs of genes linked to deletion of 1p although with a lower percentage of overlap. This list contains *Rpl5*, a gene previously linked to bortezomib resistance^38^. The overlapping gene lists may be useful to identify new candidate genes associated with the poor prognostic features of gain of 1q, deletion of 1p and 13q in patients. Regarding somatic mutations in the models, key mutations probably represent those in *Trp53* (5TGM1/5T33vv) and *Rb1* (5T2), which are also found back in patients^4,5,7,8,32,35^. Other interesting mutated genes in common with patients include *Zfhx4*, *Fgfr3*, *Fat3, Fat4, Lrp1b*, *Nf1* and *Robo1*^4,5,7,8,32,35^.

Overall, the genetic aberrations in the models target multiple pathways, including MAPK, PI3K, DNA damage and cell cycle, epigenetic regulators and extracellular regulators. In patients, these pathways are also recurrently subjected to genetic alterations^32^. Regarding MAPK and PI3K signaling, the 5T models contain genetic aberrations of genes at the receptor level and at the level of downstream mediators. This emphasizes the dysregulation of MAPK and PI3K pathways in these models for MM. The FGFR is a tyrosine kinase receptor upstream of RAS/MAPK and PI3K/AKT signaling^39^. The mutations detected here are within the extracellular IG-like domains and may affect ligand binding and/or dimerization of the receptor, thereby potentially resulting in enhanced signaling^40^. The 5T33vv model also contains a mutation in *Nf1*, a RAS GTPase-activating protein which inhibits Ras isoforms and signaling through its intrinsic GTPase activity^41^. Moreover, the genetic alterations involve several regulatory and catalytic subunits of the MAPK and PI3K pathways. How these mutations affect the activation status of the pathways has yet to be determined. Nevertheless, we did find clear differences in sensitivity towards MEK1/2 or PI3K inhibition between the models. Our findings indicate that the 5T33vv cells are dependent on both MAPK and PI3K signaling. 5T2 cells on the other hand are more dependent on PI3K signaling, while 5TGM1 is more dependent on MAPK signaling.

Patients also display mutations in TP53 and other DNA damage-related proteins such as *BRCA2, ZFHX4, ATM* and *ATR*^4,5,42^. These events are associated with poor prognosis^5,33^, and were also detected in the 5T models. Deletion of *Brca2* is a common event in the 5T models. Recently, mutations in *BRCA2* have been identified^7^, but their role remains unknown. 5T33vv and 5TGM1 cells harbor a mutation in *Trp53* in the DNA binding site, resulting in the loss of acetylation dependent apoptosis mediated by p53 upon genotoxic stress^43^. 5T33vv cells also contain a mutation in *Zfhx4*, a zinc-finger transcription factor. *Zfhx4* is important for differentiation and prevention of glioblastoma formation^44^, but its functional role in MM progression has not been investigated. Taken together, the 5T models contain several genetic defects that target key pathways known to be involved in myelomagenesis.

Regarding the mutational signatures, Walker *et al*. and Bolli *et al*. identified an APOBEC signature in agreement with the observation made by Alexandrov *et al*. who refers to signature 2. According to Walker *et al*. and Bolli *et al*., the second dominant signature results from spontaneous deamination of methylated cytosine to thymine. This is in contrast to Alexandrov *et al*. who refers to signature 5 which has an unknown etiology^5,35,45^. The dominant signature 17 in the 5T models has no known etiology. Signature 17 is found in several cancer types suggesting a rather broad mechanism with a high amount of T:A to G:C transversions linked to the improper repair of oxidized guanines^46^. Further investigation is needed to clarify whether this is linked to signature 17. An update of sequencing results in MM patients showed the presence of signature 8 and 9 in MM patients^31^. Signature 9 appears to be linked with AID, while signature 8 has an unknown cause^31^. In agreement, these two signatures are also found in the 5T models. Interestingly, we also found signature 3 in the 5TGM1 cells. This signature is linked with defective homologous recombination and BRCA1/2 mutations^45^. The three tested models all show copy number loss of *Brca2*, while no non-synonymous mutations are found in *Brca*1 or 2. Nevertheless, only 5TGM1 shows this signature. This indicates that other mechanisms besides BRCA mutations contribute to HR deficiency and the related mutational signature 3. This has also been suggested by Alexandrov *et al*.^47^. Signature 21 and 15, found in 5T2, are linked with mismatch repair deficiency (MMR) and microsatellite instability. Several components of the MMR machinery are altered in the 5T2 model due to copy number alterations. For example, *MSH2, MSH5, MSH6* and *PMS1* are amplified, while *MSH3, PMS2* and *EXO1* show copy number loss. How these changes affect mismatch repair and whether these contribute to mutational processes remains to be defined.

To conclude, the genetic landscape of 5T models is heterogeneous and overlaps with at least a subset of MM patients with respect to the affected genes and pathways. The 5T2 model displays large aneuploidy with a large similarity to gain of 1q and mutation in *Rb1*. The 5T33 and 5TGM1 models contain genetic aberrations similar to del13q in patients and a mutation in *Trp53*. These models may therefore be suitable to identify new biomarkers and investigate the associated genetic defects.

## Material and Methods

### Mice and 5TMM models

C57BL/KaLwRij and C57BL/6 mice were purchased from Envigo (Horst, The Nedtherlands). The experimental and housing methods were carried out in accordance with relevant guidelines and regulations as depicted by the Ethical Committee for Animal Experiments of the Vrije Universiteit Brussel (license no. LA1230281). All experimental protocols were approved by the Ethical Committee for Animal Experiments of the Vrije Universiteit Brussel (internal license no. 15-281-2). 5T33 and 5T2 models were maintained as previously described^48^. The 5TGM1 cell line was cultured at 37 °C and 5% CO_2_ in RPMI-1640 medium (Lonza, Basel, Switserland) supplemented with 100 U/ml penicillin/streptomycin, 1 mM Na-pyruvate, 2 mM glutamine (Gibco, Eggenstein, Germany) and 10% fetal calf serum (FCS, Hyclone, UT, USA).

### CNA detection by ultra-low coverage whole-genome sequencing

Whole-genome DNA libraries of 2 murine models, 1 cell line and 4 tails were prepared with Illumina’s TruSeq DNA sample preparation kit V2 according to the manufacturer’s instructions. Random DNA sequencing was performed on an Illumina HiSeq2500, using a V4 flow-cell generating 50 bp single end reads. Further details can be found in the supplementary methods.

### Whole-exome sequencing, mutation detection and annotation

Starting from the whole-genome libraries described above, genomic DNA libraries were prepared with Illumina’s TruSeq DNA sample preparation kit V2 according to the manufacturer’s instructions. Whole-exome capture was done using the Nimblegen SeqCap EZ Developer Library kit (110624_MM9_Exome L2R_D02_EZ_HX1_9999042611). The resulting libraries were sequenced on a HiSeq2500 (Illumina) using a V4 flow cell generating 2× 125-bp paired-end reads. The sequencing data were analyzed with our in-house developed pipeline for mouse exomes^49^. Further details can be found in supplementary methods.

### Mutational signatures

The signatures of the somatic mutations were calculated using the 3-base pair context of each mutation giving a 96-substitution classification for each subject. A conversion of our WES data was performed to be able to compare our signatures to whole genome-based signatures. We deconstructed the resulting signatures into the 30 established COSMIC somatic signatures (downloaded fall 2016). The analysis and plotting of the somatic signatures was performed using deconstructSigs^50^ and SomaticSignatures^51^, two R-packages available in Bioconductor.

### Multiple myeloma patient data

We used multiple myeloma patient data from studies published by Lohr *et al*. and Walker *et al*.^4,5^. The cohort published by Lohr *et al*. consists of 203 patients of which bone marrow biopsies were collected at institutions from the Multiple Myeloma Research Consortium (MMRC). The samples were subjected to whole-genome and whole-exome sequencing to detect mutations and copy number alterations^4^. The cohort published by Walker *et al*. consist of 463 patients enrolled in the National Cancer Research Institute Myeloma XI trial. Whole-exome sequencing was performed on the collected bone marrow biopsies^5^. The copy number alterations of the 5T models were compared to the data published by Lohr *et al*. The mutations present in the 5T models were compared to both the data published by Lohr *et al*. and Walker *et al*. The patient data was retrieved from published supplementary data files.

### Statistical analysis

To asses statistically the similarity between the murine models and human MM tumors, we calculated the overlap between genes mutated in the MM models and in the human MM datasets of Lohr *et al*.^4^ and Walker *et al*.^5^ which contain 203 and 463 patients, respectively. The 5TGM1 model was excluded since it was derived from and genetically similar with the 5T33vv model. Only murine genes with a human orthologue according to Ensembl’s Biomart browser were considered. A binomial distribution based on the total number of (possible) overlapping genes was used to calculate the significance of the overlap. Fold enrichment values were calculated by the real overlap divided by the expected overlap. For drug testing experiments, statistical analysis was done with One-way Anova followed by Tukey’s multiple comparison test. GeneE was used to summarize the presence of copy number alterations and mutations (https://software.broadinstitute.org/GENE-E/).

## Electronic supplementary material

Supplementary information

Supplementary tables

## Data Availability

The raw sequencing reads from the whole-genome and whole-exome experiments are available in the ArrayExpress database (www.ebi.ac.uk/arrayexpress) under accession E-MTAB-5302 and E-MTAB-5282, respectively. The processed data files are included as supplementary files.
