# Supplementary material for: The genetic landscape of 5T models for multiple myeloma
Source: Sci Rep. 2018 Oct 9;8:15030. doi: 10.1038/s41598-018-33396-w (PMC6177465; doi:10.1038/s41598-018-33396-w)
Supplement: Supplementary file 1 — Supplementary information [file 41598_2018_33396_MOESM1_ESM.doc]

**SUPPLEMENTARY INFORMATION**

**The genetic landscape of 5T models for multiple myeloma.**

Ken Maes^1†*^, Bram Boeckx^2†^, Philip Vlummens^1,3^, Kim De Veirman^1^, Eline Menu^1^, Karin Vanderkerken^1^, Diether Lambrechts^2††^, Elke De Bruyne^1††^

^1^ Department of Hematology and Immunology, Myeloma Center Brussels, Vrije Universiteit Brussel, Brussel, 1090, Belgium

^2^ Laboratory for Translational Genetics, Department of Oncology, Katholieke Universiteit Leuven, VIB Center for Cancer Biology, Leuven, 3000, Belgium

³ Department of clinical hematology, Ghent University Hospital, Gent, 9000, Belgium

† contributed equally

†† contributed equally

Running Title: The genetic landscape of 5TMM models.

* Corresponding author:

Dr. Ken Maes, Department of Hematology and Immunology, Myeloma Center Brussels, Vrije Universiteit Brussel, Laarbeeklaan 103, B-1090 Brussels, Belgium

tel: +32 2 477 4401

fax: +32 2 477 4405

e-mail: kemaes@vub.ac.be

**Supplementary Material and Methods**

**Purification of 5TMM cells**

5T33 inoculated mice take approximately 3 weeks to develop MM while 5T2 inoculated mice take approximately 3-4 months. We collected bone marrow from diseased mice at passage 3 by flushing the long bones. Next, BM was filtered using a nylon gause and subjected to red blood cell lysis. Samples were washed with PBS supplemented with 1% BSA and 0.01% azide. Next, cells were stained with anti-idiotype antibodies (in-house production) and anti-CD11b-FITC (ImTec Diagnostics, Antwerp, Belgium) for 30min at 4°C. Following washing with PBS/BSA/azide, the samples were incubated with the secondary antibody rat anti-mouse IgG1-APC (BD Bioscience, Erembodegem, Belgium) for 30min at 4°C. After the last washing step, cells were dissolved in PBS/BSA azide supplemented with 2µl 7AAD (BD Bioscience) per million cells and FACS sorted. To obtain >95% pure tumor populations, we gated out CD11b (because of cross-reaction of anti-idiotype antibody and myeloid cells) and 7AAD-positive cells followed by positive selection of anti-idiotype positive cells.

**DNA isolation**

DNA from 5TGM1 cells, sorted 5T33 and 5T2 cells, and mouse tails was isolated using the Wizard Genomic DNA purification kit (Promega, Madison, WI, USA). DNA was dissolved in 80µl PBS + 4µl RNAse A and incubated for 10min at 37°C. After adding proteinase K and incubation for 10min at 56°C, DNA was purified using the Qiamp DNA mini kit according to manufactures instructions. (Qiagen, Hilden, Germany) DNA was eluted in 100µl 10mM Tris and stored at -80°C for further processing.

**CNA detection by ultra-low coverage whole-genome sequencing**

Raw sequencing reads were mapped to the mouse reference genome (GRCm38/mm10) using the Burrows-Wheeler Aligner (BWA)[^1^](#_ENREF_1), and duplicate reads were removed. On average we obtained 11,443,131 reads per sample (Supplementary Table 1). These reads were binned in regions of 300 Kb windows and after correction for genomic waves with the PennCNV software package transformed into LogR values[^2^](#_ENREF_2). Subsequently these data were segmented using the Ascat algorithm. The genomic instability was quantified by the aberrant genomic fraction, which was calculated as the % of the genome with an estimated copy number <1.7 or >2.3. (Supplementary Table 1).

**Whole-exome sequencing, mutation detection and annotation**

The sequencing data were analyzed with our in-house developed pipeline for mouse exomes[^3^](#_ENREF_3). Briefly, raw sequencing reads were mapped to the mouse reference genome (GRCm38/mm10) using BWA. Duplicate reads were removed and the mapped reads were further processed with the Genome Analyser ToolKit (GATK) for realignment and base recalibration[^4^](#_ENREF_4). The tumor samples were sequenced at an average depth of 77x, having 96% of the exome covered over 10x. The germ-line samples derived from the tail of the mice were on average covered 74x, while 97% of the exome was covered over 10x. Substitutions were identified with GATK’s HaplotypeCaller and small insertions and deletions were identified by Dindel. These mutations were annotated by Annovar[^5^](#_ENREF_5). On average 5096, 4604 and 985 exonic substitutions and 220, 200 and 165 indels were called in the murine MM samples, the C57Bl/KalwRij and the C57BL/6 germ-line samples, respectively. The significant lower number of mutations in the C57Bl/6 germ-line samples is expected since the reference genome is built based on this mouse strain. Somatic events were selected by the following criteria: a) the substitutions must be present in at least 10% of the tumor reads, b) the variant quality had to be at least 30 and the variant is covered over 10x. In addition to these criteria are all indels manually reviewed in the Integrative Genome Viewer (IGV). This strategy results in 1447 substitutions and 46 indels for the 3 MM samples. The effect of the somatic mutations was assessed by PROVEAN, SIFT and GDI scores. Strain specific exonic SNPs were identified in the C57Bl/KaLwRij and C57Bl/6 strain. Only homozygous mutations were considered.

**Supplementary table information**

Supplementary table 1: Metrics of ultra-low coverage whole-genome sequencing.

Supplementary table 2: Overview of copy number alterations in the 5TMM models. Yellow highlights indicated cancer-consensus gene (Related to Figure 1 and Supplementary Figure S1).

Supplementary table 3: Overlap of CNA of the 5T models with a human dataset published by Lohr et al. (Related to Figure 1 and 5).

Supplementary table 4: Top 25 of the pathway enrichment analysis of the copy number alterations in the 5T models (Related to Figure 1).

Supplementary table 5: Metrics of exome sequencing

Supplementary table 6: Somatic substitutions and indels present in C57Bl/6 and C57Bl/KaLwRij compared to the reference genome version Mm10.

Supplementary table 7: Overview of all substitutions and indels present in the 5TMM models (Yellow highlights indicate cancer consensus genes) (Related to Figure 3).

Supplementary table 8: Top 25 of the pathway enrichment analysis of the mutations in the 5T models (Related to Figure 3).

Supplementary table 9: overview of one-way analysis of variance (Related to Figure 4).

**Supplementary Figures**

**Supplementary figure S1: Copy number alterations of cancer consensus genes in the tested 5T models.**

Shallow whole-genome sequencing was performed to identify copy number alterations in the 5T models. Red dots represent normalized read count for 300Kb bins (log R value). The green line represents segmented copy number values. Gene names of cancer consensus genes within regions with copy number alterations are provided (Related to Supplementary table 2).

**
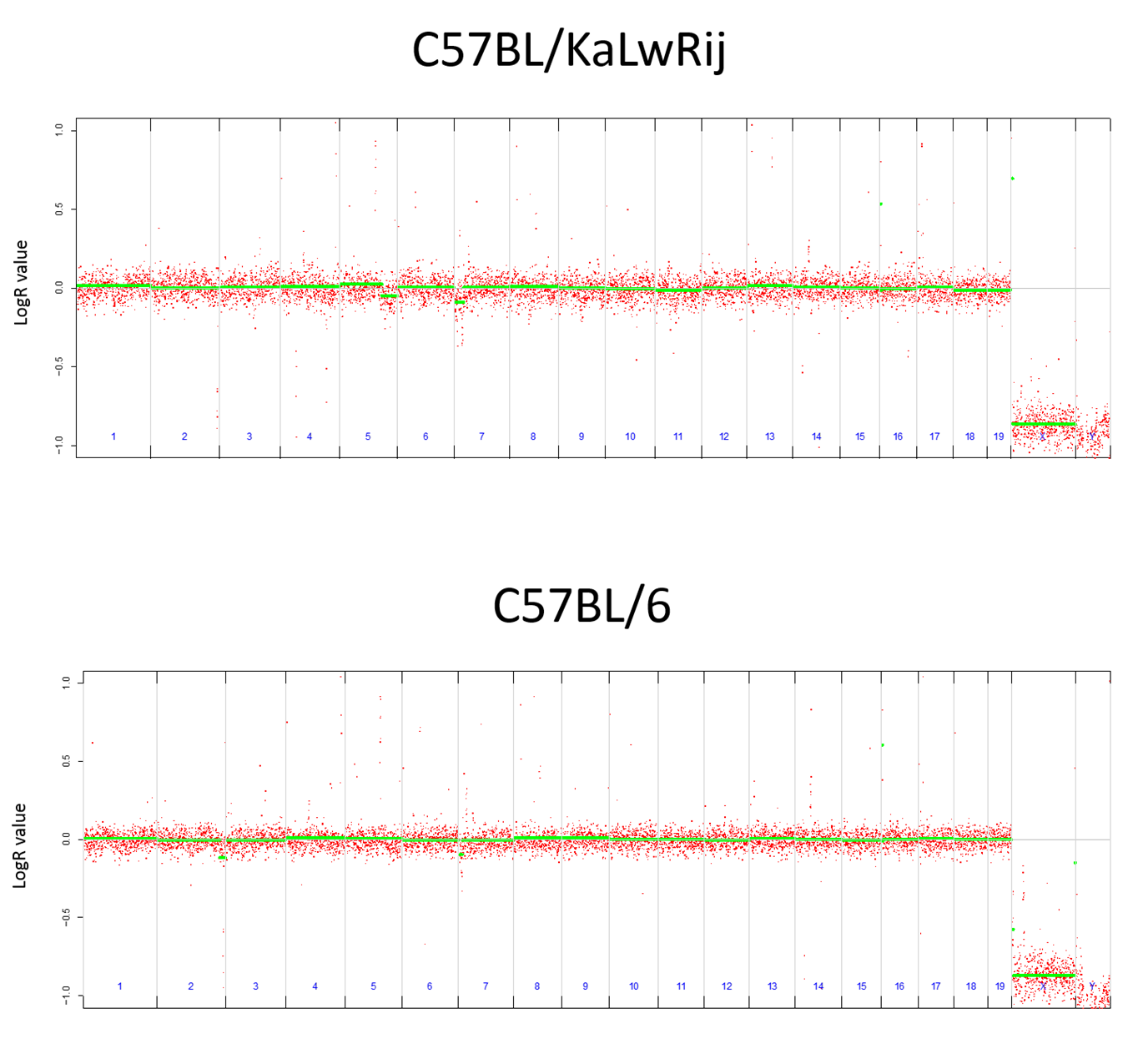
**

**Supplementary figure S2: Copy number profile of the germline DNA of C57Bl/6J and C57Bl/KaLwRij mice.** Shallow whole-genome sequencing was performed to identify copy number alterations in the mice strains C57Bl/6J and C57Bl/KaLwRij. Red dots represent normalized read count for 300Kb bins (log R value). The green line represents segmented copy number profiles.

**References:**

1 Li, H. & Durbin, R. Fast and accurate short read alignment with Burrows-Wheeler transform. *Bioinformatics* **25**, 1754-1760 (2009).

2 Wang, K. *et al.* PennCNV: an integrated hidden Markov model designed for high-resolution copy number variation detection in whole-genome SNP genotyping data. *Genome research* **17**, 1665-1674 (2007).

3 Nassar, D., Latil, M., Boeckx, B., Lambrechts, D. & Blanpain, C. Genomic landscape of carcinogen-induced and genetically induced mouse skin squamous cell carcinoma (vol 21, pg 946, 2015). *Nature Medicine* **22**, 217-217 (2016).

4 McKenna, A. *et al.* The Genome Analysis Toolkit: A MapReduce framework for analyzing next-generation DNA sequencing data. *Genome research* **20**, 1297-1303 (2010).

5 Langmead, B. & Salzberg, S. L. Fast gapped-read alignment with Bowtie 2. *Nat Methods* **9**, 357-U354 (2012).

6 Choi, Y. & Chan, A. P. PROVEAN web server: a tool to predict the functional effect of amino acid substitutions and indels. *Bioinformatics* **31**, 2745-2747 (2015).

7 Itan, Y. *et al.* The human gene damage index as a gene-level approach to prioritizing exome variants. *Proc Natl Acad Sci U S A* **112**, 13615-13620 (2015).
